# Global health education in medical schools (GHEMS): a national, collaborative study of medical curricula

**DOI:** 10.1186/s12909-020-02315-x

**Published:** 2020-10-28

**Authors:** InciSioN UK Collaborative, Soham Bandyopadhyay, Hannah S. Thomas, Binay Gurung, Isobel Trout, Shavinthi W. Wadanamby, Melika Akhbari, Karisma Sharma, J. Edward Fitzgerald, Ewen M. Harrison, Adrian D. Smith, Thomas Shortland, Rashida Patel, Roba Khundkar, Riana Patel, David Clark, Michael Dunn, Oliver Johnson, Nusrat Hussain, Dmitri Nepogodiev, Parivrudh Sharma, Shahnoor M. Amin, Pat Lok, Amir Mohammed, Catherine Dominic, Lotta Gustafsson, Abigail Jamieson, Eleanor Deane, Felicity Greenfield, Fiona Jobson, Anmol Arora, Rhys D. Wenlock, Ahmed Bilal, Maarja-Liis Ferry, Chung S. Chai, Emma Sharland, James McLaren, Beatrice Prosser, Saud Alfadhel, Agata Oliwa, Nitish Nachiappan, Muha Hassan, Connor Moore, Pedra Rabiee, Latifa Haque, Mariam Gaddah, Meltem Sarigul, Alan Penney, Won Y. Yoon, Anuradha Ponnapalli, Katarina Hoernke, Tom Poundall, Isabella Burns, Annabel Killen, Luisa Hofmaier, Arina Toma, Heather Lawson, James Bevan, Morgan Weiland, Kiana Bowden, Chiara Cotronei, Farhiya Omar, Mariam Ahmed, Jordan Cazier, Emel Yildirim, Belle Liew, Ankit Bhatt, Dilan Parmar

**Affiliations:** 1 Medical Sciences Division, University of Oxford, Oxford, UK; 2 University of Edinburgh College of Medicine, Edinburgh, UK

**Keywords:** Global health, Medical education, Medical school, Collaborative, Curricula, Diversity, Pandemic, WHO

## Abstract

**Background:**

Global health is the study, research, and practice of medicine focused on improving health and achieving health equity for all persons worldwide. International and national bodies stipulate that global health be integrated into medical school curricula. However, there is a global paucity of data evaluating the state of global health teaching in medical schools. This study aimed to evaluate the extent of global health teaching activities at United Kingdom (UK) medical schools.

**Methods:**

A national, cross-sectional study assessing all timetabled teachings sessions within UK medical courses for global health content during the academic year 2018/19. Global health content was evaluated against a comprehensive list of global health learning outcomes for medical students.

**Results:**

Data from 39 medical courses representing 86% (30/36) of eligible medical schools was collected. Typically, medical courses reported timetabled teaching covering over three-quarters of all global health learning outcomes. However, a wide degree of variation existed among granular global health learning objectives covered within the different medical courses. On average, each learning outcome had a 79% [95% CI: 73, 83%] probability of being included in course curricula. There were a number of learning outcomes that had a lower probability, such as ‘access to surgeons with the necessary skills and equipment in different countries’ (36%) [95% CI: 21, 53%], ‘future impact of climate change on health and healthcare systems’ (67%) [95% CI: 50, 81%], and ‘role of the WHO’ (54%) [95% CI: 28, 60%].

**Conclusions:**

This study served as the first national assessment of global health education and curricula within UK medical schools. Through a formalised assessment of teaching events produced by medical schools around the country, we were able to capture a national picture of global health education, including the strengths of global health prioritisation in the UK, as well as areas for improvement. Overall, it appears broad-level global health themes are widely discussed; however, the granularities of key, emerging areas of concern are omitted by curricula. In particular, gaps persist relating to international healthcare systems, multilateral global health agencies such as the WHO, global surgery, climate change and more.

## Background

Global health is the study, research, and practice of medicine focused on improving health and achieving health equity for all persons worldwide [1]. In modern-day society, where health challenges transcend national borders and governments, it is imperative that healthcare professionals (HCPs) understand and engage with global issues to improve healthcare delivery and medical practice around the world. For instance, diseases are able to cross geographical boundaries more rapidly than ever before [2] and have become an ever increasing threat to global security [3]. HCPs are key workers for mitigating and managing disease threats to maximise human safety and survival. In order to prevent and prepare for future pandemics, it is key that all HCPs are given the training to understand international disease epidemiology and trends. Equally, a better understanding of whole population health and an appreciation of the complex relationships between certain communities, ill health and health inequities is essential for effective healthcare leadership in service planning [4]. Moreover, it is the ethical responsibility of HCPs, as health advocates, to learn from one another transnationally to improve health around the world [5]. The aforementioned concepts are core principles of medical practice [6, 7]. Therefore, these professional values must be fostered and encouraged at an early stage of medical training [8].

There have been repeated calls for more robust global health education within medical training programmes from students [9–11] and faculty alike [5, 12–14]. International legislative voices, such as the World Health Organisation (WHO) now insist that global health be included in medical school curricula [15–17]. Many medical schools incorporate global health education into their curriculum through student selected (optional) modules or through intercalated Bachelor of Science degrees [6, 14]. However, global health principles have been mandated to be included in medical school curricula as essential, non-elective modules [12, 18–21]. Documents from a regulatory body for medical professionals in the United Kingdom (UK) detail specific global health learning outcomes and competencies that medical students are expected to achieve by graduation [18–20].

However, it is unclear whether medical schools have integrated global health learning outcomes into their compulsory syllabi. This is because there is a paucity of data evaluating the current state of global health teaching in medical schools. In particular, there is limited data on curricula, teaching methods and quantity of global health education outside of the United States (US) [22]. The Global Health Education in Medical Schools (GHEMS) study is a UK based, multicentre, collaborative study that aimed to characterise global health education and curricula within UK medical schools. The primary aim of this study is to formally map the extent of timetabled global health teaching activities within UK medical schools. This information can be used by medical schools and regulators to ensure that an adequate global health education is being provided to all medical students.

## Method

### Study design and participants

The study was conducted as per the previously published study protocol [23]. This was a national study evaluating global health education within UK medical school curricula during the academic year 2018/19. Medical schools were eligible to participate if they were recognised by the General Medical Council (GMC) as being able or being under review to award a UK medical degree in 2019. Data variables were based on a list of global health learning outcomes published by the Global Health Learning Outcomes Working Group (GHLOWG) [12]. This list was developed through consultations with a consortium of global health stakeholders, comprising universities, the public, the Royal Colleges, and other professional, educational, and civil society bodies, with discussions focused on the targeted outcomes listed in guidelines published by the GMC. For the purposes of this study, the list was amended following consultations with the lead author of the GHLOWG publication [12] and a board of cross-specialty advisors comprised of educationalists and global health professionals. The list was modified to improve interpretability for students and to add a learning objective (LO) related to global surgery – recently added as a mandatory LO to the national undergraduate curriculum in surgery [24] – to produce the final list of 42 LOs (Appendix 1).

Two collaborators were recruited at each participating centre by utilising existing networks of national and local bodies interested in global health. All collaborators attended a training and support session delivered by the GHEMS steering committee. Following the training session, collaborators ensured the correct departmental approval had been achieved prior to collecting data. The GHEMS steering committee maintained regular contact with all data collectors in order for collaborators to receive prompt clarifications regarding project uncertainties and provide feedback on local issues or questions raised by their institution. The authorship of this paper was modelled according to previous collaborative publications [25].

### Data collection

Collaborators obtained timetables for all curricular years of medicine at their institution for the academic year 2018/19. Separate timetables were obtained for undergraduate and graduate-entry medicine course curricula as timetabled teaching often differs between courses within the same university due to differences in course-length: undergraduate courses are typically five to six years in length, while graduate-entry courses are usually four to five years in length. Each collaborator identified global health curricular events from the timetables and independently coded them to populate a standardised data collection template formulated by the steering committee as per the study protocol (Appendix 2) [23]. Collaborators were advised not to discuss institution results with each other during the data collection phase. Following data collection, the data points in agreement (90% of all data points) between collaborators at the same institution were recorded. For the 10% of data points encountering disagreement, conflicts were resolved by mutual agreement or through a member of the GHEMS steering committee reviewing the evidence and making the final judgement. All final decisions were relayed back to the collaborators and feedback was sought from medical leadership to ensure that coding accurately represented the state of global health teaching within their medical school. Following this, permission was obtained from selected participating centres to utilise the global health content taught at their institution to create an amalgamation of frequently discussed topics (Appendix 3).

### Study variables

Variables pertaining to characteristics of the timetabled learning event – course year, undergraduate/graduate-entry course, and style of teaching - were collected. The assigned LOs for each timetabled event were recorded. The duration of time that each timetabled event spent on a global health LO was not recorded. Timetabled events for student selected modules and intercalated years were excluded from further analysis. Variables pertaining to medical elective placements at each institution were also collected, including length of the elective and the year of study. The content and location of electives are likely to be highly variable from one student to another, and as such it was beyond the scope of this study to collect more granular data about electives.

### Statistical analysis

Data was analysed using descriptive statistics to determine the proportion of LOs included in each UK medical course curriculum. For individual LOs, we calculated the proportion of medical schools reported to be delivering that LO. For groups of LOs (themes and sub-themes), we determined the median number, mean proportion, and range of LOs covered within that group across reporting schools. We used exact binomial methods to estimate 95% confidence intervals around proportions. We applied a multilevel Poisson model with robust variance to compare coverage of individual and grouped LOs to average coverage using dummy variables, modelling variance between medical schools as a random effect. We also used this approach to assess differences between LO coverage between undergraduate and graduate-entry medical courses. Where reported, confidence intervals were 95% and *p*-values were two-tailed. LOs and sub-themes that were covered by fewer than 80% of participating centres have been highlighted, and divided into those that are covered by some medical courses (60–80%), and those that are covered by few medical courses (< 60%).

### Ethical considerations

Advice on ethical approval was sought from King’s College London Research Ethics Committee and Oxford Medical Sciences Inter-Divisional Research Ethics Committee. Both committees stated this study did not require ethical approval.

### Role of the funder

The author(s) received no financial support for the research, authorship and/or publication of this article.

## Results

### Characteristics of timetabled teaching sessions

**Fig. 1:**
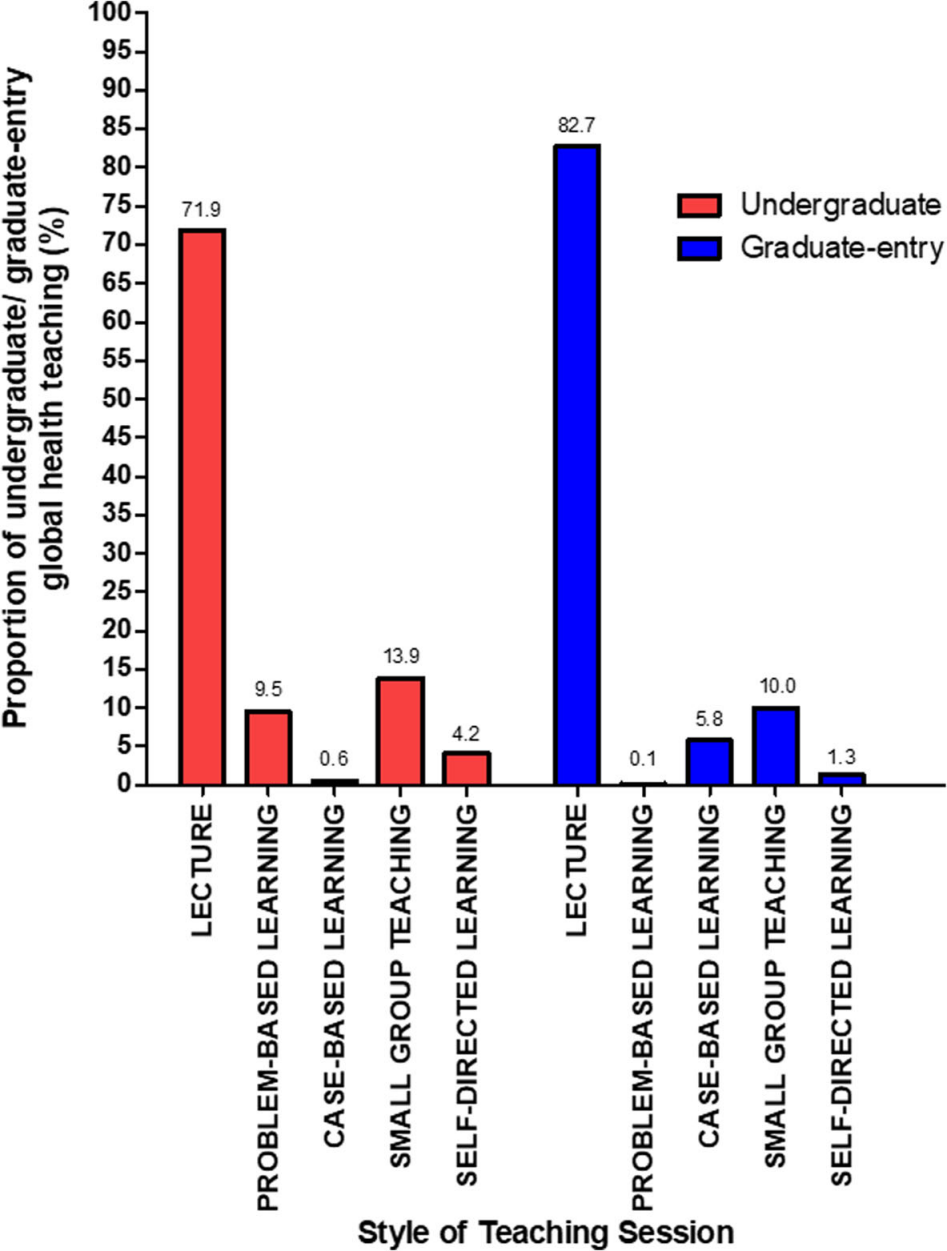
Teaching styles employed by medical courses to deliver content of global health learning objectives

Eighty-three percent of eligible UK medical schools were represented in this study (30/36). Eligible centres were excluded due to an inability to recruit collaborators or a lack of permission granted by the prospective institution. Among consenting institutions, 23 were located in England, five in Scotland, two in Wales and none in Northern Ireland. Data was extrapolated based on the type of medical course offered within the institution: undergraduate and/or graduate-entry course. Overall, data from 39 medical courses was collected: 28 undergraduate medical courses and 11 graduate-entry medicine courses. A range of teaching styles were employed by each medical course to deliver LO content. Undergraduate and graduate-entry courses had a similar distribution of pedagogical techniques (Fig. 1). The majority of teaching (74%) was delivered via lectures in both undergraduate (72%) and graduate-entry (83%) courses. The remainder of the teaching was provided via small group teaching (13%; undergraduate:14%; graduate-entry: 10%), problem-based learning (8%; undergraduate:9%; graduate-entry: 0.1%), case-based learning (2%; undergraduate:1%; graduate-entry:6%), and self-directed learning (4%; undergraduate:4%; graduate-entry:1%).

### Coverage of Global Health LOs by UK medical courses

**Fig. 2:**
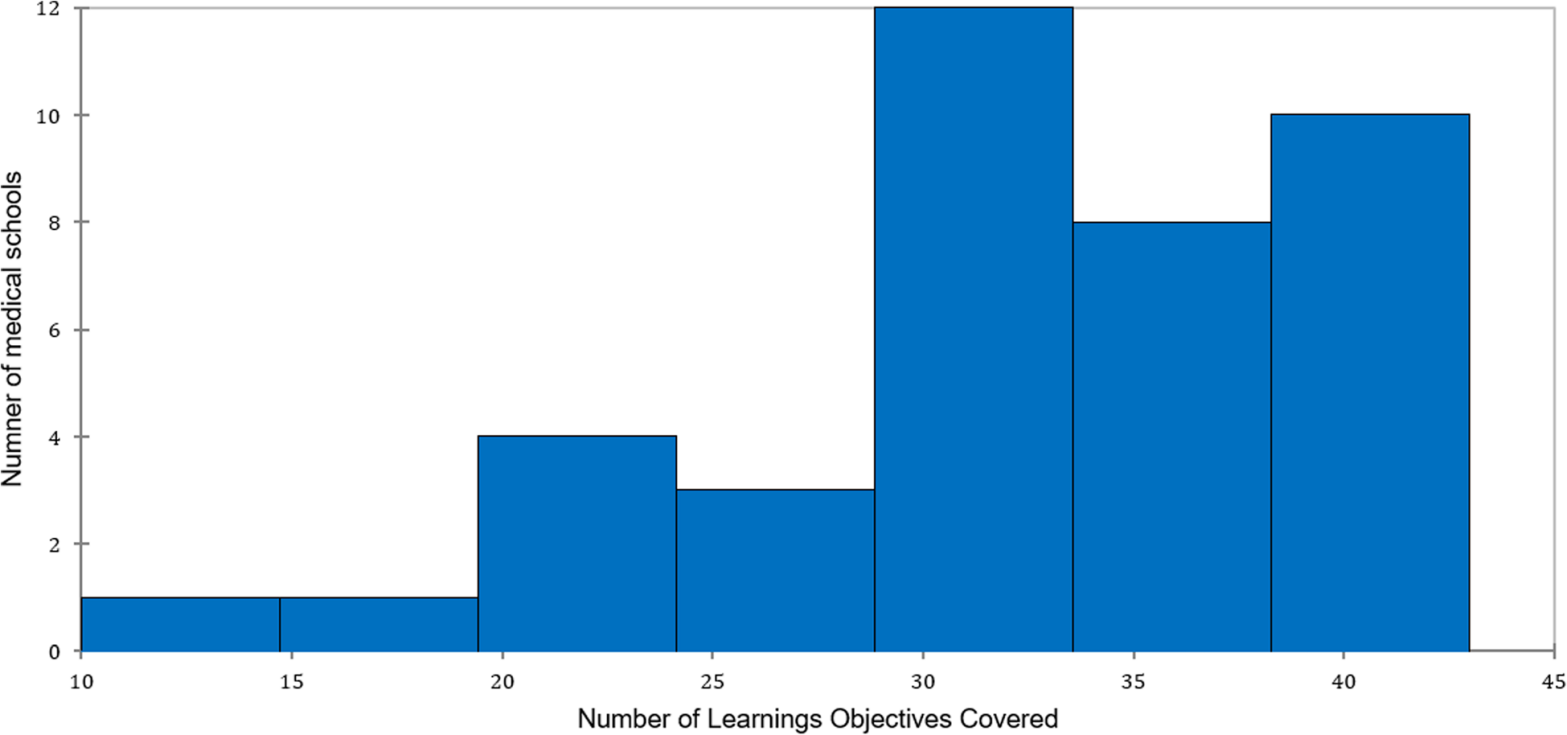
Histogram of the number of learning objectives (max 42) covered by medical schools in the UK

A total of 38 (38/39, 97%), 17 (17/39, 44%), and four (4/39, 10%) medical courses reported that they taught all global health themes, sub-themes, and LOs, respectively. Overall, medical courses taught the majority (15/17, 88%) [95% CI: 87, 95%] of global health sub-themes. The mean number of global health objectives incorporated into all timetabled teaching of any one medical course was 33/42 (79%) [95% CI: 73, 83%]. This mean was skewed left due 2 medical courses covering fewer than 20 LOs, as shown in Fig. 2. There was considerable variation with regards to the number of learning objectives covered by medical courses within each theme and sub-theme (Table 1). The greatest disparity of mean LO coverage was between the themes of global burden of disease (89%) [95% CI: 86, 92%] and organisation of health services (64%) [95% CI: 60, 68%].

**Table 1:** Learning objectives taught by medical courses divided by theme and sub-theme

Theme				Sub-Theme			
	Number of LOs	Median number of LOs covered by medical courses (range)	Mean % of LOs covered by medical courses (95% CI)		Number of LOs	Median number of LOs covered by medical courses (range)	Mean % of LOs covered by medical courses (95% CI)
Global Burden of Disease	11	10 (6–11)	89.3 (86.0–92.4)	The Health of Populations	6	6 (2–6)	91.9 (87.6–95.0)
				Migration and Disease	3	3 (1–3)	87.2 (79.7–92.6)
				Pandemics	2	2 (0–2)	84.6 (74.7–91.8)
Socioeconomic, Cultural, and Environmental conditions	6	5 (2–6)	83.8 (78.4–88.2)	Effects of violence and war on health^a^	1	1 (0–1)	61.5 (44.6–76.6)
				Health inequity	1	1 (1–1)	100 (91.0–100)
				Socioeconomic Factors affecting health	1	1 (1–1)	100 (91.0–100)
				Political Factors affecting health	1	1 (0–1)	89.7 (75.8–97.1)
				Environmental and occupational hazards and ways to mitigate their effects	1	1 (0–1)	84.6 (69.4–94.1)
				Future impact of climate change on health and healthcare systems^a^	1	1 (0–1)	66.7 (49.8–80.9)
				Political Factors affecting health	1	1 (0–1)	89.7 (75.8–97.1)
Organisation of health services	13	8 (2–13)	63.9 (59.6–68.1)	Health Systems^a^	5	4 (1–5)	70.8 (63.8–77.0)
				Workforce^a^	4	3 (0–4)	64.1 (56.0–71.6)
				Global Governance^b^	4	2 (0–4)	55.1 (47.0–63-1)
Human Rights and Ethics	7	6 (0–7)	76.9 (71.5–81.8)	Law and Ethics^a^	2	2 (0–2)	78.2 (67.4–86.8)
				Human Rights	2	2 (0–2)	83.3 (73.2–90.8)
				Vulnerable groups^a^	3	2 (0–3)	71.8 (62.7–79.7)
Cultural Diversity and Health	5	5 (0–5)	86.7 (81.1–91.1)	Communication	3	3 (0–3)	83.8 (75.8–89.9)
				Health determinants	2	2 (0–2)	91.0 (82.3–96.3)

Note: *LOs* learning objectives. ^a^Sub-themes that are covered by some medical courses (60–80%). ^b^Sub-themes that are covered by few medical courses (< 60%)

### Theme one: global burden of disease

**Fig. 3:**
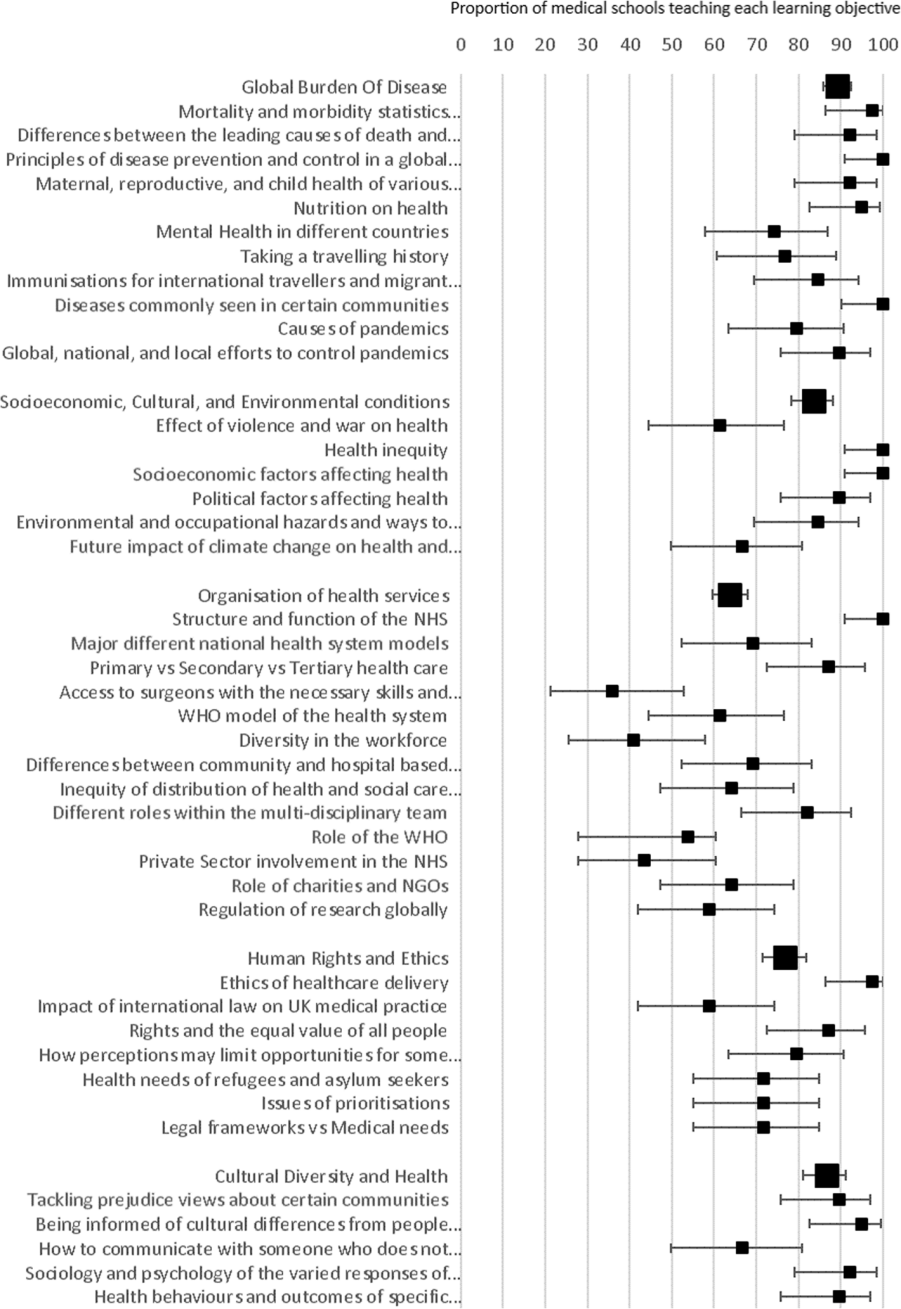
Proportion of medical schools that covered each learning objective, grouped by learning objective and theme. The average proportion of learning objectives covered by medical courses for each theme is also shown; this is indicated by the larger black boxes. Error bars indicate the 95% confidence interval

All medical courses (*n* = 39) included timetabled content pertaining to the ‘global burden of disease’ theme. Nested within this theme, the following three sub-themes: ‘the health of populations’, ‘migration and disease’ and ‘pandemics’ were offered within the teaching of 100% (39/39), 100% (39/39), and 90% (35/39) of medical courses, respectively. The three sub-themes were further subdivided into 11 global health LOS that were included in 89% (35/39) [95% CI: 74, 96%] of medical courses. Moreover, all medical courses documented the inclusion of ‘principles of disease prevention and control in a global setting’ and ‘diseases commonly seen in certain communities’ into timetabled teaching (Fig. 3 and Table 2). In addition, ‘mortality and morbidity statistics between countries’ and ‘nutrition on health’ was covered by all undergraduate medical courses and all graduate-entry medical courses, respectively. There was a significant difference between the proportion of undergraduate medical courses that reported they had scheduled teaching on ‘global, national, and local efforts to control pandemics’ (27/28,96%) and the proportion of graduate-entry medical courses that reported they had timetabled teaching on this LO (8/11, 73%) (*p* = 0.0326).

**Table 2:** The proportion of medical schools that have teaching sessions related to each learning objective

LO	% of medical courses that taught LO (95% CI)
	THEME
**Global Burden Of Disease**	
Mortality and morbidity statistics between countries	97.4 (86.5–99.9)
Differences between the leading causes of death and disability in different countries	92.3 (79.1–98.4)
Principles of disease prevention and control in a global setting	100 (91–100)
Maternal, reproductive, and child health of various countries	92.3 (79.1–98.4)
Nutrition on health	94.9 (82.7–99.3)
Mental Health in different countries^a^	74.4 (57.9–87)
Taking a travelling history^a^	76.9 (60.7–88.9)
Immunisations for international travellers and migrant communities	84.6 (69.5–94.1)
Diseases commonly seen in certain communities	100 (90.1–100)
Causes of pandemics^a^	79.5 (63.5–90.7)
Global, national, and local efforts to control pandemics	89.7 (75.8–97.1)
**Socioeconomic, Cultural, and Environmental conditions**	
Effect of violence and war on health^a^	61.5 (44.6–76.6)
Health inequity	100 (91–100)
Socioeconomic factors affecting health	100 (91–100)
Political factors affecting health	89.7 (75.8–97.1)
Environmental and occupational hazards and ways to mitigate their effects	84.6 (69.4–94.1)
Future impact of climate change on health and healthcare systemsv	66.7 (49.8–80.9)
**Organisation of health services**	
Structure and function of the NHS	100 (91–100)
Major different national health system models^a^	69.2 (52.4–83.0)
Primary vs Secondary vs Tertiary health care	87.2 (72.6–95.7)
Access to surgeons with the necessary skills and equipment in different countries^b^	35.9 (21.2–52.8)
WHO model of the health system^a^	61.5 (44.6–76.6)
Diversity in the workforce^b^	41 (25.6–57.9)
Differences between community and hospital-based staff^b^	69.2 (52.4–83.0)
Inequity of distribution of health and social care professionals^a^	64.1 (47.2–78.8)
Different roles within the multi-disciplinary team	82.1 (66.5–92.5)
Role of the WHO^b^	53.9 (27.8–60.4)
Private Sector involvement in the NHS^b^	43.6 (27.8–60.4)
Role of charities and NGOs^a^	64.1 (47.2–78.8)
Regulation of research globally^b^	59 (42.1–74.4)
**Human Rights and Ethics**	
Ethics of healthcare delivery	97.4 (86.5–99.9)
Impact of international law on UK medical practice^b^	59 (42.1–74.4)
Rights and the equal value of all people	87.2 (72.6–95.7)
How perceptions may limit opportunities for some people^a^	79.5 (63.5–90.7)
Health needs of refugees and asylum seekers^a^	71.8 (55.1–85.0)
Issues of prioritisations^a^	71.8 (55.1–85.0)
Legal frameworks vs Medical needs^a^	71.8 (55.1–85.0)
**Cultural Diversity and Health**	
Tackling prejudice views about certain communities	89.7 (75.8–97.1)
Being informed of cultural differences from people from that culture	94.9 (82.7–99.4)
How to communicate with someone who does not speak English^a^	66.7 (49.8–80.9)
Sociology and psychology of the varied responses of groups and societies to disease	92.3 (79.1–98.4)
Health behaviours and outcomes of specific backgrounds	89.7 (75.8–97.1)

Note: *LOs* learning objectives. ^a^LOs that are covered by some medical courses (60–80%). ^b^LOs that are covered by few medical courses (< 60%)

### Theme two: socio-economic, cultural, and environmental conditions

All medical courses documented inclusion of the theme ‘socio-economic, cultural, and environmental conditions’ as part of timetabled teaching. The six sub-themes that formed this broader theme: ‘effects of violence and war on health’, ‘health inequity’ and ‘socioeconomic factors affecting health’, ‘political factors affecting health’, ‘environmental and occupational hazards and ways to mitigate their effects’ and ‘future impact of climate change on health and healthcare systems’ were included in timetabled learning opportunities of 62% (24/39), 100% (39/39), 100% (39/39), 90% (35/39), 85% (33/39), and 67% (26/39) of courses, respectively (Fig. 3 and Table 2). On average, all six global health LOs within this theme were reported to be included in the timetabled teaching of 85% (33/39) [95% CI: 67, 100%] of medical courses.

### Theme three: organisation of health services

All medical courses included timetabled content pertaining to the ‘organisation of health services’ theme within their curricula. The three sub-themes embedded within this broader theme: ‘health systems’, ‘workforce’ and ‘global governance,’ were reported to be incorporated into scheduled teaching of 100% (39/39), 95% (37/39), and 85% 33/39) of medical courses, respectively. On average, each of the 13 global health LOs nested under this theme were included in the timetabled learning opportunities of 64% (25/39) [95% CI: 55, 77%] of medical courses. This mean was skewed right by the fact that all medical courses documented the inclusion of content pertaining to the ‘structure and function of the NHS’ into teaching (Fig. 3 and Table 2). Three LOs were not included in any teaching sessions by the majority (> 50%) of medical courses: access to surgeons with the necessary skills and equipment in different countries (14/39, 36%) (*p* = 0.003), diversity in the workforce (16/39, 41%) (*p* = 0.009), and private sector involvement in UK healthcare (17/39, 44%) (*p* = 0.015).

### Theme four: human rights and ethics

Ninety-seven percent of medical courses (38/39) reported that they covered the theme of ‘human rights and ethics’ as part of timetabled teaching. Embedded within this theme, the following three sub-themes: ‘law and ethics’, ‘human rights’ and ‘vulnerable groups’ were included within the learning opportunities of 97% (38/39), 92% (36/39), and 90% (35/39) of medical courses, respectively. On average, each of the seven global health LOs that form the theme of ‘human rights and ethics,’ was reported to be included in the scheduled teaching of 77% (30/39) [95% CI: 65, 88%] of medical courses (Fig. 3 and Table 2). This mean was skewed left by the relatively few medical courses that had timetabled teaching about ‘the impact of international law on UK medical practice’ (23/39, 59%). Of note, all graduate-entry medical courses included ‘ethics of healthcare delivery’ into timetabled teaching.

### Theme five: cultural diversity and health

Ninety-seven percent of medical courses (38/39) documented inclusion of the theme ‘global burden of disease’ within timetabled teaching. The two sub-themes that formed this broader theme: ‘communication’ and ‘health determinants’ were reported to be incorporated into the timetabled teaching of 97% (38/39), and 92% (36/39) of all medical courses, respectively. On average, each of the five global health LOs embedded within the umbrella theme, was reported to be included within scheduled learning opportunities of 87% (34/39) [95% CI: 73, 100%] of medical courses (Fig. 3 and Table 2). This mean was skewed left by the relatively few medical schools that had timetabled teaching about ‘how to communicate with someone who does not speak English’ (26/39, 67%). Of note, all graduate-entry medical courses reported inclusion of ‘being informed of cultural differences from people from that culture’, ‘sociology and psychology of the varied responses of groups and societies to disease’, and ‘health behaviours and outcomes of specific backgrounds’ into their timetabled teaching.

### Medical electives

The average length of elective opportunities offered at each UK medical course was 6.8 weeks. The length of the elective opportunities ranged from four weeks to ten weeks. Overall, 13 medical schools (43%, 13/30) – offering undergraduate and/or graduate entry medical courses – reported compulsory pre-elective timetabled teaching for medical students, with topics ranging from appropriate vaccinations to respecting local cultures. Two medical schools held post-elective face-to-face reflection sessions with students.

## Discussion

This study affirms that the majority of learning outcomes produced by the GHLOWG are being taught as part of timetabled teaching within the majority of UK undergraduate and graduate-entry medical courses. The pedagogical method utilised by the majority of the teaching sessions are lectures. Broad-level themes and sub-themes are well-covered within curricula; however, there is considerable variation when it comes to more specific global health LO opportunities. For instance, while all medical courses delivered teachings on the structure and function of the National Health Services (NHS), broader discussions surrounding healthcare systems and the dynamic involvement of the private and charitable sectors were neglected. Similarly, it was estimated that only over half of medical courses provided teachings on the WHO’s framework and role, leaving much to speculate regarding students’ education of international global health surveillance and security. Further, few medical courses included content on highly topical global health issues, such as global surgery and climate change. Trends were largely similar between undergraduate and graduate-entry courses; however, graduate-entry courses were less likely to cover content pertaining to pandemics. Finally, although medical schools offered approximately seven weeks of elective time, there were minimal learning opportunities pertaining to elective preparation or debriefing.

By gaining a greater understanding into global health topics exposed to UK medical students, we were able to ascertain the key lessons being instilled in the future NHS workforce. The inconsistent coverage of certain LOs between medical schools, indicates distinct differences in standards of global health education across the UK. Translational topics including awareness of cultural differences, the ethics of healthcare delivery and diseases commonly seen in certain migratory communities, are universally applicable to all students’ future careers as NHS clinicians. However, less frequently discussed topics such as healthcare systems, global health security and surveillance, global surgery and climate change also necessitate attention in order to ensure these needs do not go unmet [26, 27]. In particular, the emergence of the novel coronavirus pandemic reinforces these essential global health lessons. A thorough understanding of not just the hierarchy of the NHS, but also the structure and functioning of international healthcare systems affords students a greater opportunity to understand global health security and pandemic preparedness [28]. When the Ebola epidemic spread across West Africa, there was an acute need for medical professionals to adopt less traditional roles and new knowledge. A renewed spotlight was placed on the need for comprehensive health systems models, with integrated global health security [29]. Our study noted that few medical courses included teaching about the WHO, marking a major gap in education surrounding global multilateral health agencies. The WHO plays a vital role in preventing and addressing disease around the world, and many argue that the coronavirus pandemic has reinforced the importance of this role [30]. Overall, as future clinicians, medical students are in the unique position to harness the lessons of pandemic response to step into professional roles that mitigate disease threats [31]. Developed by the American Medical Association (AMA), health systems science (HSS) is a novel approach designed to challenge medical curricula to include regular, interdisciplinary, health systems teaching [32]. Trialled by a few medical schools in the US, HSS calls for timetabled emphasis on healthcare policy, health information technology, systems thinking and more [32]. Authors of the HSS movement caution that such reform must be accompanied by addressing student-perceived barriers such as the basic science-focused design of formal medical assessments and additional stressors [33].

In addition to the above global health needs, emerging topics such as global surgery and climate change were covered infrequently by medical curricula. In 2015, the Lancet Commission on Global Surgery released a landmark report detailing the needs of five billion people who lack access to safe, timely and affordable surgical care [34]. Advocates reinforced surgery as an “indivisible, indispensable part of health care,” with moral and economic arguments for future investment by nations [34]. At a higher-tier in the UK, the Royal Colleges have affirmed their commitment to addressing inequities in surgical care, through dynamic collaborations with the College of Surgeons of East, Central and Southern Africa (COSECSA) and SURG-Africa, among others [35]. Moreover, global surgery has been ratified by the Royal College of England as an essential topic among surgical undergraduate curriculum [24]. With parallel calls for strengthening global surgery education at the trainee-level, learning opportunities pertaining to surgical inequities are crucial for inclusion in medical school curricula [36]. Similarly, as literature continues to emerge detailing the links between climate change and health, such issues must be discussed among the future health workforce [37]. Global temperature shifts, wildfires, changing tides, disrupted animal habitats and more, challenge the homeostasis of our existing disease patterns, including the presentation of novel infectious diseases. Given the topic relevance, medical students must be equipped with climate change knowledge, in order to serve as advocates and counsel for patient wellbeing [38]. The One Health approach may serve as a blueprint for schools looking to further consider the complex effect of human, animal and environmental health on emerging disease threats [39]. Finally, elective preparation and debriefing is an area of need that has been identified by GHEMS and others [11]. With 90% of UK medical students participating in a medical elective abroad, it is particularly important to ensure students are equipped with the necessary knowledge, awareness and cultural humility to meaningfully work in settings of varied resource-levels as well as appropriately learn about other cultures and medical systems [40]. Given medical electives are a valuable opportunity for many students to consolidate and contextualise their global health teaching, it is a significant missed opportunity that only 40% of medical schools provide a mandatory timetabled preparatory course.

The GHEMS study comprehensively captured data from 83% of eligible medical schools in the UK. This has been possible through collaboration amongst students. Such collaborative projects which are driven for students, by students, have been validated internationally and have a proven record of generating robust data [25, 41]. Amongst participating medical schools, approximately 90% of centres had two collaborators independently coding all timetabled global health learning events, thereby increasing the reliability of the data. The consistency and reproducibility of the data was ensured by providing centralised training to collaborators and publishing an open access comprehensive study protocol [23]. Through the GHEMS study, local results were disseminated to institutional leadership to facilitate ongoing discussions surrounding educational quality improvement. This crucial link in the audit loop, ensures that participating centres can benefit from study results and identify strategies for global health education reform at their respective institution. Moreover, our study is timely, as several new medical schools (including the Universities of Sunderland, Lincoln and Edgehill) are currently developing their curricula. In addition, as the world combats a novel infectious disease pandemic, concerns arise surrounding the under-representation and variance of key LOs within medical school curricula. This warrants further investigation into the reasoning behind curricula development. Similar research has been conducted in both the US and Canada through formalised organisations such as the Global Health Education Consortium [22]. National recommendations have also yielded a diverse range of global health learning opportunities among American medical schools [11], suggesting the need for greater international reform of global health education. The results of GHEMS serve to guide schools in developing comprehensive curricula that addresses all recommended global health LOs. Finally, it is interesting to note that most global health LOs are being taught via lectures: the most base form of information transfer; these LOs are topics that lend themselves to in depth discussions between students. The paucity of small group teaching around global health may be an indicator that there has not been sufficient emphasis on global health to make it more of a “skill” than just knowledge.

The cross-sectional nature of our study restricted the depth of gathered curricular information. A continual audit cycle of global health teaching across multiple years would reveal the true nature of global health curricular progression within medical schools. In addition, data collection was limited to timetabled information found within institutional virtual learning environments and schedules. In this regard, authors were unable to ascertain whether planned teaching matched actual learning opportunities delivered to students. Although data pertaining to the length of each teaching session was collected, we believe this was not representative of the breadth of nuance of coverage afforded to each LO. For example, while a topic may have been listed within the learning points of a lecture, we were unable to ascertain what proportion of the total lecture time was spent on this area of interest. It is also important to acknowledge the various opportunities for external global health exposure afforded to medical students through summer projects, conferences and more. Such opportunities may depend on factors such as geographic region, local global health faculty and institutional global health funding, but nevertheless should be assessed in order to capture the variability of student experiences. Finally, this study did not evaluate institutional global health examination standards. Teaching without assessment will generally be viewed as non-compulsory, particularly by busy medical students, therefore efforts in this discipline should be directed towards robust global health educational examination as well.

## Conclusion

This study serves as the first national assessment of global health education and curricula within UK medical schools. Through a formalised assessment of teaching events produced by medical schools around the country, we were able to capture a national picture of global health education, including the strengths of global health prioritisation in the UK, as well as areas for improvement. Overall, it appears broad-level global health themes are widely discussed; however, the granularities of key, emerging areas of concern are omitted by curricula. In particular, gaps persist relating to international healthcare systems, multilateral global health agencies such as the WHO, global surgery, climate change and more. To facilitate local strengthening of global health educational opportunities, collaborators reported local results to institutional leadership to initiate discussions regarding avenues for satisfaction of the topical gaps in content identified. With models such as GHEMS, we encourage national advocacy bodies and other stakeholders to conduct ongoing status reviews of compulsory global health teachings within their medical schools, with a view to highlight areas for improvement. To equip medical students in the UK with the knowledge to operate, thrive and care for communities in our ever-globalised world, medical schools must vigilantly upgrade current curricula to reflect expert-driven recommendations for global health education. Finally, we perceive and hope that the GHEMS framework will be replicated internationally in order to address the paucity of global health educational literature in medical schools and strengthen the opportunities afforded to students to learn, grow and serve future patient populations.

## Data Availability

The datasets used and analysed during the current study are available from the corresponding author on reasonable request.

## Appendix 1

### Complete List of Global Health Themes, Sub-Themes, and Learning Objectives

The full list of forty-two global health learning objectives is listed below. The forty-two global health learning objectives can be divided into seventeen sub-themes, which fit into five global health thematic elements. Each learning objective, sub-theme, and thematic element was cross-referenced with the GHLOWG learning outcome that it relates to.

**Table:**

Thematic element (**), Sub-theme (*), and Learning objective	GHLOWG learning outcome
Global burden of disease**	Global burden of disease
1. The Health of Populations*	1. Discuss communicable and non-communicable disease at the global level.
1.1 Access to surgeons with the necessary skills and equipment in different countries	1.4 Describe the major control and prevention initiatives for communicable and non-communicable diseases that exist at the global level. (Part of the national undergraduate curriculum in surgery)
1.2 Mortality and morbidity statistics between countries	1.1 Examine and use the key measures of mortality and morbidity to compare the disease burden between regions.
1.3 Differences between the leading causes of death and disability in different countries	1.2 Describe the leading causes of death and disability at the global level as well as the anticipated trends over time. 1.3 Compare and contrast the causes of death and disability between regions and indicate why these variations exist.
1.4 Principles of disease prevention and control in a global setting	1.4 Describe the major control and prevention initiatives for communicable and non-communicable diseases that exist at the global level.
1.5 Maternal, reproductive, and child health of various countries	1.5 Explain the impact of maternal, reproductive and child health on the global burden of disease.
1.6 Nutrition on health	1.6 Explain the impact of poor nutrition on health from a global perspective.
1.7 Mental Health in different countries	1.7 Explain the importance of mental ill health as a major contributor to the burden of disease worldwide.
2. Migration and Disease*	2. Discuss the impact of international travel and migration on the diseases seen in the UK.
2.1 Taking a travelling history	2.1 Take an appropriate travel history and recognise common causes of illness in a returning traveller.
2.2 Immunisations for international travellers and migrant communities	2.3 Discuss the basis for the use of immunisations for international travellers and migrant communities in the UK.
2.3 Diseases commonly seen in certain communities	2.2 Discuss the aetiology, clinical presentation and management of diseases linked to migration, basing judgement on clinical evidence rather than prejudicial assumption. 19. Demonstrate understanding that culture is important and may influence behaviour, while acknowledging the dangers of assuming that those from a particular social group will behave in a certain way
3. Pandemics*	3. Discuss the causes and control of global epidemics.
3.1 Causes of pandemics	3.1 Identify the causes of global epidemics.
3.2 Global, national, and local efforts to control pandemics	3.2 Discuss how pandemics should be controlled at the global, national and local levels.
Socio-economic, cultural, and environmental conditions**	Socio-economic, cultural, and environmental conditions
4. Effects of violence and war on health* (also a learning objective)	1.8 Explain the importance of violence and injuries to the global burden of disease.
5. Health inequity* (also a learning objective)	5. Examine how health can be distributed unequally within and between populations in relation to socially defined measures.
6. Socioeconomic factors affecting health* (also a learning objective)	4. Demonstrate awareness of the non-clinical determinants of health, including social, political, economic, environmental and gender disparities.
7. Political factors affecting health* (also a learning objective)	4. Demonstrate awareness of the non-clinical determinants of health, including social, political, economic, environmental and gender disparities.
8. Environmental and occupational hazards and ways to mitigate their effects* (also a learning objective)	4. Demonstrate awareness of the non-clinical determinants of health, including social, political, economic, environmental and gender disparities. 6. Describe how the environment and health interact at the global level 6.1 Explain how the environment can impact on health, such as through air pollution, flooding and heat waves.
9. Future impact of climate change on health and healthcare systems* (also a learning objective)	4. Demonstrate awareness of the non-clinical determinants of health, including social, political, economic, environmental and gender disparities. 6. Describe how the environment and health interact at the global level 6.1 Explain how the environment can impact on health, such as through air pollution, flooding and heat waves. 6.2 Explain the existing and potential future impact of climate change on health and discuss ways to mitigate the effects, both at the individual and collective levels.
Organisation of health services**	Health Systems
10. Health systems*	8. Recognise that health systems are structured and function differently across the globe.
10.1 Structure and function of the NHS	8.2 Describe the structure and function of the NHS.
10.2 Major different national health system models	8.1 Describe the major different national health system models.
10.3 Primary vs Secondary vs Tertiary health care	8.4 Discuss the relevance of primary health care to health system models
10.4 WHO model of the health system	7. Discuss the essential components of a health system, using the WHO model
11. Workforce*	9. Recognise that the NHS has an international workforce and explain the impact of this within the UK and overseas.
11.1 Diversity in the workforce	9.1 Discuss the relevance of an international workforce on national standards and interprofessional communication.
11.2 Differences between community and hospital-based staff	10.3 Examine the causes and scale of inequalities in health workforce distribution that exist between community-based and hospital-based care
11.3 Inequity of distribution of health and social care professionals	10.1 Examine the causes and scale of inequalities in health workforce distribution that exist between regions and countries 10.2 Examine the causes and scale of inequalities in health workforce distribution that exist between urban and rural areas
11.4 Different roles within the multidisciplinary team	10. Examine the causes and scale of inequalities in health workforce distribution that exist 21. Work effectively with colleagues from different ethnic, religious and social backgrounds.
12. Global governance*	Global health governance
12.1 Role of the WHO	12. Discuss the role of the WHO as the international representative body of national governments for health. 12.1 Describe the functions of the WHO concerning international health policy, disease surveillance, data collection, sharing best practice and setting international norms.
12.2 Private Sector involvement in UK healthcare	8.3 Discuss the involvement of multinational corporations and foreign health systems in delivering health care to UK patients. 11. Demonstrate awareness of the complexity of global health governance, including the roles of international organisations, the commercial sector and civil society.
12.3 Role of charities and NGOs	8.3 Discuss the involvement of multinational corporations and foreign health systems in delivering health care to UK patients. 11. Demonstrate awareness of the complexity of global health governance, including the roles of international organisations, the commercial sector and civil society.
12.4 Regulation of research globally	13. Discuss how health-related research is conducted and governed globally. 13.1 Recognise that research trials are subject to rules and guidelines that have been set at the international level. 13.2 Explain how the processes of drug research, development and patenting can impact health and access to medicines
Human rights & ethics**	Human rights & ethics
13. Law and Ethics*	15. Examine how international legal frameworks impact on health care delivery in the UK. 20.2 Identify potential ethical concerns relating to the use of family members as translators.
13.1 Ethics of healthcare delivery	16.2 Discuss and critique how the concept of a right to health impacts on health care delivery in the UK. 20.2 Identify potential ethical concerns relating to the use of family members as translators.
13.2 Impact of international law on UK medical practice	15. Examine how international legal frameworks impact on health care delivery in the UK. 13.2 Explain how the processes of drug research, development and patenting can impact health and access to medicines 17.2 Recognise that vulnerable groups are protected by specific legal frameworks.
14. Human rights*	14. Respect the rights and equal value of all people without discrimination and provide compassionate care for all. 17.2 Recognise that vulnerable groups are protected by specific legal frameworks.
14.1 Rights and the equal value of all people	16. Discuss and critique the concept of a right to health. 16.1 Discuss the definition of a human right. 14.1 Respect patient values and beliefs relating to their health, treatment and end of life care.
14.2 How perceptions may limit opportunities for some people	14. Respect the rights and equal value of all people without discrimination and provide compassionate care for all. 14.1 Respect patient values and beliefs relating to their health, treatment and end of life care.
15. Vulnerable groups*	17. Describe the particular health needs of vulnerable groups and migrants.
15.1 Health needs of refugees and asylum seekers	17.1 Describe the key health needs of refugees, asylum seekers and undocumented migrants in the UK from biomedical, psychological and social perspectives, and how these change over time
15.2 Issues of prioritisations	18. Discuss the role of doctors as advocates for their patients, including the importance of prioritising health needs over other concerns and adhering to codes of professional conduct.
15.3 Legal frameworks vs Medical needs	15. Examine how international legal frameworks impact on health care delivery in the UK. 13.2 Explain how the processes of drug research, development and patenting can impact health and access to medicines 17.2 Recognise that vulnerable groups are protected by specific legal frameworks.
Cultural diversity and health**	Cultural diversity and health
16. Communication*	20. Communicate effectively with those from different ethnic, religious and social backgrounds, where necessary using external help. 20.3 Conduct a consultation and examine patients, demonstrating sensitivity to different backgrounds.
16.1 Tackling prejudice views about certain communities	2.2 Discuss the aetiology, clinical presentation and management of diseases linked to migration, basing judgement on clinical evidence rather than prejudicial assumption. 19. Demonstrate understanding that culture is important and may influence behaviour, while acknowledging the dangers of assuming that those from a particular social group will behave in a certain way
16.2 Being informed of cultural differences from people from that culture	19. Demonstrate understanding that culture is important and may influence behaviour, while acknowledging the dangers of assuming that those from a particular social group will behave in a certain way
16.3 How to communicate with someone who does not speak English	20.1 Describe how to access external help for translation, including translation services and leaflets in an appropriate language, and recognise how this can impact communication. 20.2 Identify potential ethical concerns relating to the use of family members as translators. 20.3 Conduct a consultation and examine patients, demonstrating sensitivity to different backgrounds.
17. Health determinants*	19. Demonstrate understanding that culture is important and may influence behaviour, while acknowledging the dangers of assuming that those from a particular social group will behave in a certain way
17.1 Sociology and psychology of the varied responses of groups and societies to disease	19. Demonstrate understanding that culture is important and may influence behaviour, while acknowledging the dangers of assuming that those from a particular social group will behave in a certain way 19.2 the doctor-patient relationship
17.2 Health behaviours and outcomes of specific backgrounds	19.1 health-seeking behaviour 19.3 the use of alternative medicines and treatments 19.4 lifestyle and substance misuse 20.4 Access information about the impact of a specific background on health risks.

## Appendix 2

### Data Collection Template

Below is a sample of our data collection template. Each row represented a documented teaching session, while each column represented a variable of interest regarding that teaching session. The start of the form collected baseline data pertaining to the key characteristics of the teaching session, while later components were comprised of each learning objective subdivided by theme (e.g. Global Burden of Disease) and sub-theme (e.g. The Health of Populations). Collaborators identified “Yes” or “No” if the teaching sessions fulfilled or did not fulfil the learning objective.

## Appendix 3

Amalgamation of Key Topics Covered in Global Health Education within UK Medical Schools. Topics were derived from the written learning outcomes reported by collaborators for each teaching session. Common themes were synthesised across schools to create an amalgamation of frequently covered topics. This could serve as the beginning of a framework for integrating global health learning objectives into existing compulsory timetabled teaching sessions.

**Table:**

Global Health Topics	Topics that can be integrated into existing timetabled sessions
Epidemiology	• Epidemiology of communicable diseases • Epidemiology of non-communicable diseases • Mortality statistics at a national/ international level • Morbidity statistics at a national/ international level
Health of certain population demographics	• Vulnerability to disease • Health of refugees and asylum seekers • Health issues of minority groups • Exploring the factors behind travelling families being less likely to access healthcare opportunities
Public Health	• Defining public health • Preventing Disease • Population level interventions e.g. vaccinations • Health economics and resource allocation • Disease burden • Changing population demographics • Sustainability as a concept • Provision of contraception • Role of the media • Role of surgery and anaesthesia • Role of the police • Role of social services • Street level bureaucracy • Sanitisation • Role of Millennium Development Goals • Screening programs
Health Systems	• Organisation of services within a healthcare system • Purpose of the NHS • Funding of healthcare systems i.e. public vs private • Service provision • Current and future challenges facing healthcare • Clinical governance • Communication between services within a system • Role of power in policy process • Primary vs Secondary vs Tertiary • Complaints procedures
Determinants of health	• Regional differences in service provision • Genetics • Nutrition • Migration • Social standing • Prejudice against the disabled • Prejudice against the aged • Prejudice against certain cultures • Prejudice against certain religions • Prejudice against certain genders • Prejudice against certain sexualities • Prejudice against certain ethnicities • Impact of being in a certain profession • Impact of certain behaviours on health • Impact of violence • Ease of access to services and trained staff e.g. surgeons • Advantages of wealth • Living conditions • Educational attainment • Adherence to treatment
WHO	• Declaration of Alma-Ata • International prioritisation • Checklist • Sustainable development goals • Health system strategy • Action on the social determinants of health • Global Action Plan
Organisations involved in healthcare independent from any government	• Gates Foundation • UNICEF • Doctors of World • Role of charities • Aid vs trade
Roles of healthcare professionals	• Health promotion • Communicating appropriately with patients, especially those who are vulnerable • Understanding how to balance patient priorities with clinical decisions • Taking a travel and dietary history • Be involved in research and quality improvement projects • Work in a multidisciplinary team • Act within your competency • Knowledge of ICER and QALYs • Educators • Knowledge of risk and how to convey it to patients • Importance of collaborating within and between professions • Recognise own prejudices and assumptions that perpetuate disadvantages • Respectful curiosity and empathy of people from other cultures • Understand the role of protocols • Recognise modern slavery and human trafficking
Ethics	• End of life care • Capacity • Principle of justice • Safeguarding • Euthanasia • Prejudice • Abortion • Genetics • Data storage • Autonomy • Confidentiality • Research • Medical Elective • Human rights • Organ and tissue retention and use for transplants
Laws	• Mental Capacity Act • Mental Health Act • Deprivation of liberties • Abortion • End-of-life • Equality Act • Taxation • Disability Discrimination Act • Child Protection • Impact on health • Human fertilisation and embryology act • Using laws and taxes to modify behaviour • EU working time directive • Declaration of Helsinki • Female genital mutilation laws
Patient perspectives	• Impact of stigma • Impact of stereotyping • Importance of appreciating the cultural background of a patient • Role of complementary and alternative medicine • Need for professional interpreters where language is a barrier • Importance of doctor-patient relationships • Impact of illness on patient and their family • Sources of support for patients • Importance of their values being considered • Issues faced by the poor • Issues faced due to social isolation • Implications of organ selling
Impact of violence	• Health of domestic abuse victims • How to manage victims of knife crime • Gender-based violence • Impact of violence on children • Impact of female genital mutilation • Sexual violence and its impacts
Politics and health	• Implications of Brexit on the NHS • Bridging research and policy • Shaping the way care is provided • Economics as a driving force for political decisions • Humanitarian aid
Environment and health	• Impact of climate change on health • Effects on development • Impact of pollutants on health • Natural disasters
Communicable diseases	• Epidemiology • Methods of prevention • Methods of spread • Methods of controlling spread • Surveillance • Outbreaks • Recognising symptoms • Antimicrobial resistance
Non-communicable diseases	• Long-term impacts • Recognising symptoms • Impact of the environment • Impact of political decisions • Impact of socio-economic factors • Changes in trend as the demographic changes
Child heath	• Aetiology of failure to thrive • Safeguarding • Nutrition • Weaning practices • Development screening • Effect of the family on the health of the child • Effect of the society on the health of the child • Emotional health • Implications for future health
Nutrition	• Risk factors for obesity • Importance of protected mealtimes • Under-nutrition causes and management • Challenge of providing adequate nutrition for the whole global population • Water scarcity as a health issue • Composition of a healthy diet • Consequences of hunger and starvation
Mental health	• Key issues across the world • Bias surrounding mental health • Impact of stigma • Mental health issues predominant in each gender • Service delivery • Cultural differences in the symptoms exhibited for mental illnesses • Impact of physical disease on mental health • Anxiety over medical procedures
Woman’s health	• Antenatal care • Pregnancy complications • Post-partum complications e.g. fistula • Barriers to care • Impact of female genital mutilation • Impact of displacement for maternal health

## Abbreviations

AMA: American Medical Association
COSECSA: College of Surgeons of East, Central and Southern Africa
GHEMS: Global Health Education in Medical Schools study
HCPs: Healthcare professionals
HSS: Health systems science
LO: Learning Outcome
NHS: National Health Services
UK: United Kingdom
US: United States
WHO: World Health Organisation
